# Innovative Energy Storage Smart Windows Relying on Mild Aqueous Zn/MnO_2_ Battery Chemistry

**DOI:** 10.1002/advs.202402369

**Published:** 2024-05-29

**Authors:** Hamid Palamadathil Kannattil, Lluis Martinez Soria Gallo, Kenneth D. Harris, Benoît Limoges, Véronique Balland

**Affiliations:** ^1^ Université Paris Cité, CNRS Laboratoire d'Electrochimie Moléculaire Paris F‐75013 France; ^2^ National Research Council Canada – Nanotechnology Research Centre Edmonton AB T6G 2M9 Canada; ^3^ Department of Mechanical Engineering University of Alberta Edmonton AB T6G 2G8 Canada

**Keywords:** aqueous battery, electrochromic battery, reversible metal electrodeposition, reversible zinc electrodeposition

## Abstract

Rechargeable mild aqueous Zn/MnO_2_ batteries are currently attracting great interest thanks to their appealing performance/cost ratio. Their operating principle relies on two complementary reversible electrodeposition reactions at the anode and cathode. Transposing this operating principle to transparent conductive windows remains an unexplored facet of this battery chemistry, which is proposed here to address with the development of an innovative bifunctional smart window, combining electrochromic and charge storage properties. The proof‐of‐concept of such bifunctional Zn/MnO_2_ smart window is provided using a mild buffered aqueous electrolyte and different architectures. To maximize the device's performance, transparent nanostructured ITO cathodes are used to reversibly electrodeposit a high load of MnO_2_ (up to 555 mA h m^−2^ with a CE of 99.5% over 200 cycles, allowing to retrieve an energy density as high as 860 mA h m^−2^ when coupled with a zinc metal frame), while flat transparent FTO anodes are used to reversibly electrodeposit an homogenous coating of zinc metal (up to ≈280 mA h m^−2^ with a CE > 95% over 50 cycles). The implementation of these two reversible electrodeposition processes in a single smart window has been successfully achieved, leading for the first time to a dual‐tinting energy storage smart window with an optimized face‐to‐face architecture.

## Graphical abstract

This study provides the proof‐of‐concept of single‐ and dual‐tinting energy‐storage smart windows relying on the reversible and concomitant electrodeposition of MnO_2_ and zinc on two transparent electrodes in a mild aqueous electrolyte, and discusses the associated performance and challenges.

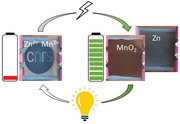

## 1 Introduction

Integrating reversible electrochromic properties with reversible charge storage in a single bi‐functional device is an appealing concept and a currently‐booming research topic.^[^ ^1^, ^2^, ^3^ ^]^ The concept builds on the simple fact that electrochromic windows and electrochemical charge storage devices share a similar sandwich structure, relying on a face‐to‐face assembly of two current collectors separated by an electrolyte and exploiting the electrochemical reactivity of an active material on each side.^[^ ^4^ ^]^ The concept of bifunctional electrochromic charge storage devices was initially proposed for monitoring the state‐of‐charge of a supercapacitor,^[^ ^5^ ^]^ and thereafter, it was rapidly extended to the development of so‐called energy storage smart windows (ESSWs).^[^ ^6^ ^]^ These ESSWs represent a new generation of smart windows, with significant advantages over conventional electrochromic windows which require an energy supply for both coloration and bleaching.^[^ ^7^ ^]^ ESSWs not only save energy by controlling the transmission of solar irradiation into building, but they furthermore restitute the energy stored during their coloring or bleaching process. ESSWs can therefore be energy‐neutral, and even better, they can be used as temporary storage units for locally‐produced energy from renewable sources, thereby increasing the energy autonomy of buildings.^[^ ^8^ ^]^

The development of these ESSWs, however, requires a paradigm shift compared to conventional smart windows, insofar as coloration efficiency, switching time, and cyclability are no longer the only criteria to be considered. Energy density and Coulombic efficiency also become important parameters.^[^ ^9^ ^]^ In addition, to meet the demanding requirements of eco‐sustainability and societal acceptability, the development of devices exploiting green energy storage chemistries and operating in safe mild aqueous electrolytes has to be favored.^[^ ^10^ ^]^ This has naturally led to the development of mild aqueous ESSWs relying on a zinc anode.^[^ ^7^, ^11^ ^]^ Indeed, zinc is a particularly advantageous negative electrode in aqueous batteries due to its abundance, low cost, high gravimetric capacity, and low reduction potential (E° = −0.76 V vs NHE).^[^ ^12^, ^13^ ^]^ When operating in a weakly acidic electrolyte, zinc is reversibly electrodeposited from its soluble oxidized form, Zn^2+^, according to the following reversible electrochemical reaction:^[^ ^14^ ^]^

(1) $$Zn^{2+}+2\;e^{-}⇄\;\mathrm{Zn}$$

Rechargeable mild aqueous Zn‐based batteries are currently attracting a great deal of attention, because of their potential to meet the requirements of large scale energy storage.^[^ ^13^, ^15^ ^]^ This has inspired a number of groups to develop mild aqueous Zn‐based electrochromic batteries.^[^ ^11^ ^]^ In these ESSW devices, the reversible zinc electrodeposition (RZE) typically takes place at an opaque frame or belt of zinc metal, offset from the light transmission paths.^[^ ^16^, ^17^, ^18^, ^19^, ^20^, ^21^, ^22^, ^23^, ^24^, ^25^ ^]^ As a result, the electrochromic features rely solely on the active material at the cathode, with the added drawback of leading to inhomogeneous electric field lines,^[^ ^26^ ^]^ resulting in color gradients.^[^ ^16^, ^18^, ^19^ ^]^ This later also raises the question of scalability to large window sizes. In an attempt to address these issues, semi‐transparent anodes made of zinc pre‐coated fine metal mesh grids^[^ ^27^, ^28^ ^]^ or Ni@Ag nanofibers deposited on a transparent substrate^[^ ^29^ ^]^ were proposed. However, these approaches remain relatively complex to implement and thus poorly suited to the development of large scale windows. Performing RZE directly on the surface of a transparent electrode is certainly a better option. It not only reduces complexity during device manufacturing, but also enables the development of large size dual‐tinting Zn‐based ESSWs combining two electrochromic windows face to face. Such an architecture will benefit from the coloring efficiency of reversible metal electrodeposition (RME) to achieve full opacity and neutral color over a wide wavelength range,^[^ ^30^ ^]^ while ensuring homogeneous coloring and improving the cyclability of large‐scale devices thanks to a better spatial distribution of the electric field.^[^ ^26^ ^]^ However, this topic is still in its infancy, and RZE on transparent electrodes has only recently been investigated by Barile's group.^[^ ^31^, ^32^, ^33^ ^]^ The benefit of zinc electrodeposition for color‐neutral smart windows with enhanced heat‐shielding capabilities was clearly established, but cyclability is limited due to the co‐precipitation of zinc (hydr)oxides, leading to a rapid loss of optical contrast.^[^ ^31^ ^]^ Despite these efforts, no bi‐functional device taking advantage of the reversible electrodeposition of zinc at a transparent anode in combination with an electrochromic cathode has been reported.

Regarding the cathode, only a few electrochromic materials have been paired with a zinc anode in electrochromic batteries. Among them, most are defined as cathodic electrochromic materials, meaning that they color upon reduction, leading to self‐coloring devices.^[^ ^16^, ^17^, ^20^, ^21^, ^22^, ^23^ ^]^ However, high potential anodic electrochromic materials, coloring upon oxidation, are preferred for the design of efficient ESSWs capable of reversibly storing locally‐produced energy (by a renewable energy source such as a solar panel). This scenario allows light flux to be attenuated in the daytime upon device charge, and the associated stored energy to be released whilst restoring the transparent state in the absence of sunlight. Fulfilling these conditions are the Prussian blue (PB) derivatives, which have been successfully used to design aqueous Zn‐based ESSWs.^[^ ^18^, ^19^, ^28^ ^]^ By exploiting the redox transition between the colored PB charged state and the transparent Prussian white (PW) discharged state (associated with reversible cation intercalation in the PB film), these devices are capable of delivering voltages of ≈1.24 V upon discharge while recovering transparency. Because they initially incorporate a colored PB cathode, they require a discharge step to restore their transparency, and therefore zinc must also be present at the anode when devices are initially assembled. At present, the best performance ESSWs in terms of charge storage was reported by Wang et al. for a Zn/PB device, exhibiting an areal energy density close to 80 mA h m^−2^ at 0.07 A m^−2^.^[^ ^19^ ^]^ However, this device fails to reach full opacity over a large wavelength range and remains semi‐transparent in its charged state.

Manganese dioxide is another interesting candidate to be used at the cathode side. There are only a few examples of studies reporting electrochromic properties of MnO_2_ films, and this material has rarely been proposed for electrochromic devices, probably due to its modest coloration efficiency (≈20 cm^2^ C^−1^ @ 500 nm).^[^ ^34^ ^]^ However, MnO_2_ has experienced renewed interest for the development of low cost and sustainable aqueous batteries,^[^ ^35^ ^]^ and its use in the design of rechargeable electrochromic Zn‐ion batteries is emerging.^[^ ^24^ ^]^ Regarding the charge storage mechanism, it has been recently demonstrated that MnO_2_ undergoes a 2‐electron proton‐coupled electrodissolution/electrodeposition to/from soluble Mn^2+^ in mild aqueous buffered electrolytes, according to the following reversible electrochemical reaction:^[^ ^36^, ^37^ ^]^

(2) $$\mathrm{Mn}O_{2}+2\;e^{-}+4\;\mathrm{AH}⇄{\mathrm{Mn}}^{2+}+4\;A^{-}+2\;H_{2}O$$

where AH and A^−^ stand for the acidic and basic forms of the buffer. As a result, in a mild aqueous buffered electrolyte, a colored MnO_2_ film can reversibly electrodeposit on a transparent electrode.^[^ ^36^ ^]^ MnO_2_, therefore, has the potential to be an effective cathodic electrochromic material, suitable for coupling with RZE at the anode to design innovative Zn/MnO_2_ ESSWs with improved charge storage performance. To avoid pH gradients at the interfaces and precipitation of metal (hydr)oxides during cycling, the use of buffered aqueous electrolytes is to be preferred for such assembly.^[^ ^38^ ^]^ Moreover, as this ESSW concept features two complementary reversible electrodeposition reactions, the device has the advantage of being directly assembled in the bleached discharged state, with all the active materials being present in the aqueous electrolyte in the form of solvated and uncolored Zn^2+^ and Mn^2+^ ions. This has several advantages. First, it reduces the production costs of smart windows, since the active material no longer has to be pre‐deposited on the transparent conductive window. In addition, surface capacity and coloration of the device can be easily adjusted by the operator, and rejuvenation strategies can also be developed to improve cycle life (vide infra).

The present work aims to integrate the Zn/MnO_2_ battery chemistry into innovative ESSWs. Devices featuring different architectures were built and characterized. We first focused on an architecture involving the reversible electrodeposition of MnO_2_ at a transparent nanostructured cathode in combination with RZE at a zinc frame metal anode. The resulting single‐tinting ESSW is demonstrated to lead in significantly improved charge storage performance as compared to the previously proposed Zn‐based ESSWs. Next, we constructed the first dual‐tinting Zn/MnO_2_ ESSW, by achieving simultaneously the reversible electrodeposition of Zn and MnO_2_ at transparent electrodes arranged face to face. The challenges and issues associated with this specific architecture are highlighted, and a simple strategy for improving the device's cyclability is presented.

## 2 Results and Discussion

### 2.1 MnO_2_ Electrochromic Cathode for High Energy Density ESSW

Transparent nanostructured indium tin oxide (ITO) electrodes prepared by glancing angle deposition (GLAD, see Experimental Section for details) were used to achieve the reversible electrodeposition of MnO_2_ from an aqueous electrolyte containing a Mn^2+^ salt.^[^ ^39^ ^]^ The GLAD technique is a one‐step vapor deposition method, allowing the growth of an array of ITO nanocolumns perpendicular to the flat, underlying substrate (i.e., commercial ITO on glass) with high control over the film morphology, thickness and porosity. These electrodes were selected because of their high electroactive surface area, allowing reversible electrodeposition of large amounts of MnO_2_ despite its intrinsically low electronic conductivity.^[^ ^37^ ^]^ To ensure high porosity, and thus easy access of the electrolyte throughout the entire GLAD‐ITO film thickness, the deposition angle was fixed at 85°, and the film thickness was tuned from 1 to 2.5 µm. For sake of convenience, these electrodes of varying thickness are hereafter referred as ITO_1_, ITO_1.6_ and ITO_2.5_. After thermal annealing steps to improve the conductivity and transparency of the GLAD‐ITO deposit (see Experimental Section), the electrodes were characterized by SEM (Figure S1, Supporting Information) and electrochemistry. These analyses show that the electroactive surface area of the GLAD‐ITO electrodes, inferred from their electrical double layer capacitance in cyclic voltammetry, increases linearly with the film thickness (Figure S2, Supporting Information). These electrodes were used as the cathode in a first smart window assembly, as depicted in Figure S3 (Supporting Information), combining a zinc metal frame as the anode, silicone frames as separators, and a glass slide as the back window. The devices, referred to as Zn/ITO_x_ with x = 1, 1.6, or 2.5, were filled with ≈1.2 mL of a mild buffered aqueous electrolyte (pH 4.8) made of 1 m acetate buffer along with 0.1 m MnCl_2_ and 0.1 m ZnCl_2_. It is now well‐established that such an acetate‐buffered electrolyte allows for reversible electrodeposition of MnO_2_ upon oxidation, according to the following electrochemical reaction:^[^ ^36^, ^37^, ^38^, ^40^ ^]^

(3) $$\begin{array}{c} {\mathrm{Mn}}^{2+}+2\;H_{2}O+4\;{\mathrm{CH}}_{3}\mathrm{CO}O^{-}\;⇄\mathrm{Mn}O_{2}+2\;e^{-}+4\;{\mathrm{CH}}_{3}\mathrm{COOH} \end{array}$$

This reaction has previously been extensively studied by us on various conductive substrates,^[^ ^36^, ^37^, ^40^ ^]^ and the electrodeposited MnO_2_ characterized by numerous techniques, demonstrating that MnO_2_ is present in an amorphous phase with an average oxidation state of ≈3.8.

The transmittance spectra of the devices are given in Figure S3 (Supporting Information). It can be clearly seen that the overall transmittance of the Zn/ITO_1_ and Zn/ITO_1.6_ devices remains high enough for electrochromic applications, whereas the transmittance of the Zn/ITO_2.5_ device is limited in the visible range, due mainly to diffuse scattering. Accordingly, this Zn/ITO_2.5_ assembly was not further used in the present work.

**Figure 1:**
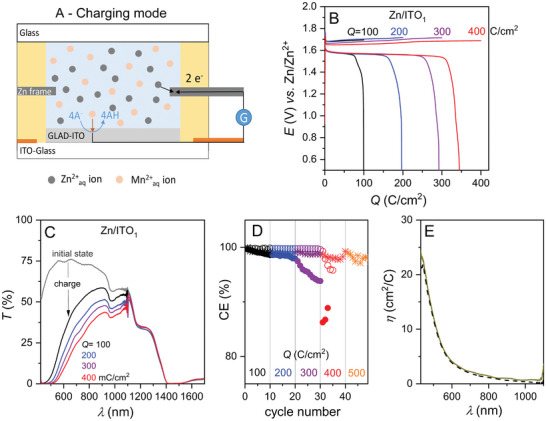
Operating principle and performances of Zn/ITO devices. A) Coloration /charging principle of the Zn/ITO devices. AH and A^−^ refer to acetic acid and acetate, while the symbol G refers to the galvanostatic charge process. B) Continuous galvanostatic charge/discharge cycles performed at a Zn/ITO_1_ device (rate: 0.3 mA cm^−2^) in a 1 m acetate buffer containing 0.1 m MnCl_2_ and 0.1 m ZnCl_2_ (only the first of the ten cycles is shown). The load was initially set at (black data) 0.1 C cm^−2^ and then incremented by 0.1 C cm^−2^ every ten cycles (the 0.2 C cm^−2^ is shown in blue, 0.3 C cm^−2^ in violet, 0.4 C cm^−2^ in red). C) Transmittance spectra of the Zn/ITO_1_ device recorded at the end of the galvanostatic charges in A, with in grey the transmittance spectrum of the as‐fabricated device. D) Coulombic efficiency of the (filled circles) Zn/ITO_1_, (empty circles) Zn/ITO_1.6_ and (stars) Zn/ITO_2.5_ devices, including data (orange) for a load up to 0.5 C cm^−2^. E) Coloration efficiency as a function of the wavelength calculated for a charge of 0.1 C cm^−2^ for the (black dashed) Zn/ITO_1_ and (dark yellow) Zn/ITO_1.6_ device.

The Zn/ITO_1_ and Zn/ITO_1.6_ smart windows were then subjected to galvanostatic cycling, starting with a load of 0.1 C cm^−2^ during the first ten charges, which was then stepwise incremented by 0.1 C cm^−2^ every ten cycles. Under such conditions, reversible MnO_2_ electrodeposition at the cathode (Equation 3) is coupled with reversible zinc electrodeposition (Equation 1) at the zinc metal frame anode (see the operating principle in **Figure** **1**A). As a result, the full device operates through the following global reversible reaction:

(4) $$\begin{array}{ccc} {\mathrm{Zn}}^{2+} & + & {\mathrm{Mn}}^{2+}+2\;H_{2}O+4\;{\mathrm{CH}}_{3}\mathrm{CO}O^{-}⇄\mathrm{Zn}\\ & & +\;\mathrm{Mn}O_{2}+4\;{\mathrm{CH}}_{3}\mathrm{COOH} \end{array}$$

The potentiometric charge/discharge curves and transmittance spectra simultaneously recorded during the first galvanostatic cycle of the Zn/ITO_1_ device are shown in black in Figure 1. The galvanostatic cycles are characterized by highly stable charge and discharge potentials (Figure 1B), low‐voltage hysteresis (≈0.11 V), and high Coulombic efficiencies (CE > 99%) (Figure 1C). At the end of the charging steps, the transmittance of the device steadily decreases for *λ* < 1000 nm (Figure 1C), and similar results were observed for the Zn/ITO_1.6_ device (Figure S5, Supporting Information). The coloration efficiency *η* (in cm^2^ C^−1^) of both devices was calculated over the entire wavelength range according to the following expression:

(5) $$\eta =\frac{\log\left(T_{discharged}/T_{charged}\right)}{Q}$$

where *T_discharged_* and *T_charged_* are the transmittance of the device in the fully discharged and charged states, respectively, and *Q* (in C cm^−2^) is the total amount of charge passed during the coloration/charging step. The corresponding curves plotted in Figure 1E overlap nicely for the two devices, indicating that the coloration efficiency only relies on reversible MnO_2_ electrodeposition, without significant contribution from the nanostructured GLAD‐ITO film. At 500 nm, the value of *η* = 12 cm^2^C^−1^ is close to the values previously obtained for MnO_x_ films,^[^ ^34^ ^]^ but it drops to 3 cm^2^ C^−1^ at 633 nm, a wavelength commonly used to monitor the optical contrast of electrochromic devices involving a PB cathode. It is worth recalling here that coloration efficiency is a less critical parameter for ESSWs compared to conventional electrochromic devices, for which high coloration efficiency is required to reduce the power consumption upon cycling between colored and bleached states. Since the amount of charge reversibly stored in an ESSW device will be much greater than that (consumed) by a conventional electrochromic window, intense coloring, or even total opacity, can still be achieved with materials characterized by low coloring efficiency. This is even desirable if coloring is to be precisely modulated according to the amount of charge.

To assess the maximum capacity of MnO_2_ that can be electrodeposited with high reversibility, the charge capacity was stepwise increased by 0.1 C cm^−2^ every ten cycles (which corresponds to an incremental time increase of 333 s and MnO_2_ mass load of ≈54 µg cm^−2^).^[^ ^36^ ^]^ It is be note here that the zinc metal anode acts as an almost infinite reservoir of zinc, capable of supplying zinc indefinitely upon discharge, irrespective of the efficiency or zinc electrodeposition upon charge, so we assume that the Coulombic efficiency of the whole device (Figure 1D) is controlled by the reversibility of the MnO_2_ electrodeposition at the cathode. We observe that the CE of the Zn/ITO_1_ device (solid dots in Figure 1D) decreases slightly upon cycling for charges up to 0.2 C cm^−2^ and degrades more significantly once the charge is set at 0.3 C cm^−2^ or higher. In contrast, the Zn/ITO_1.6_ device exhibits a high and stable CE value, up to a charge of 0.3 C cm^−2^ (see empty dots in Figure 1D), and only begins to decrease significantly once the charge reaches 0.4 C cm^−2^ (equivalent to a MnO_2_ load of ≈216 µg cm^−2^).^[^ ^36^ ^]^ We attribute this difference to the higher electroactive surface of the ITO_1.6_ electrode, which allows for improved reversible electrodeposition of MnO_2_.^[^ ^37^ ^]^ This was further confirmed upon cycling the Zn/ITO_2.5_ device under identical conditions, which could be cycled with high CE and no color accumulation for higher loads (Figure 1E).

**Figure 2:**
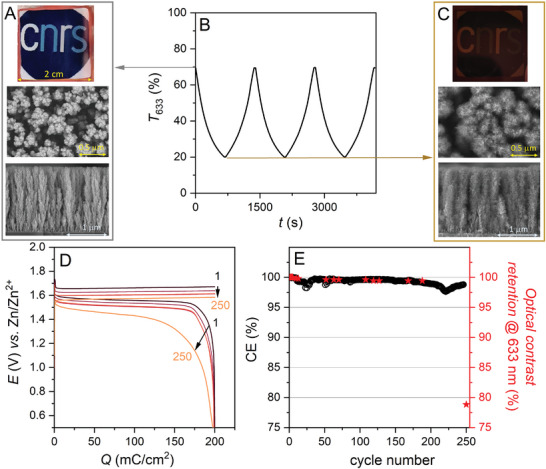
A,C) Photographs of the Zn/ITO_1.6_ smart window in the (A) discharged and (C) first charged states. Middle and bottom: top‐view and cross‐sectional SEM images of the GLAD‐ITO_1.6_ electrode before and after electrodeposition of MnO_2_ up to 0.2 C cm^−2^. B) Transmittance monitored at 633 nm during the three initial galvanostatic charge/discharge cycles performed at a Zn/ITO_1.6_ device (rate: 0.3 mA cm^−2^) in a 1 m acetate buffer containing 0.1 m MnCl_2_ and 0.1 m ZnCl_2_ and or a load set at 0.2 C cm^−2^. D) From dark red to orange: galvanostatic charge/discharge cycles 1, 50, 100, 200, and 250. E) (black dots) Coulombic efficiency and (red stars) optical contrast retention over 250 cycles.

Consequently, the Zn/ITO_1.6_ device was selected for long‐term cycling, setting the areal capacity at 0.2 C cm^−2^ (equivalent to 555 mA h m^−2^), the rate at 0.3 mA cm^−2^, and thus the charging time at 667 s. Note that the coloration time is inversely proportional to the intensity, and that it can therefore be reduced by increasing the intensity, as we will see later. The homogeneity of the deposit at the macroscale was confirmed by the homogeneous brown coloration of the electrode upon first charge (**Figure** **2**), while *ex‐situ* SEM analysis of the charged electrode confirms the deposition of MnO_2_ throughout the intercolumnar pores of the GLAD‐ITO film, with a carambola‐shaped morphology as expected for this mode of deposition,^[^ ^36^, ^37^ ^]^ without extending beyond the thickness of the GLAD film.

A selection of galvanostatic charge/discharge profiles recorded over 250 cycles are shown in Figure 2D. The first cycle, centered on an average voltage *E*_av_ = 1.61 V, is characterized by a discharge potential of 1.56 V versus Zn/Zn^2+^, allowing a total energy density of 860 mW h m^−2^ to be retrieved during discharge. It is also characterized by a low hysteresis of 0.11 V and a CE as high as 99.99%, leading to an overall energy efficiency (EE) of 93.4%. Regarding the electrochromic performances, the device presents an optical contrast of 61% at 500 nm and 51% at 633 nm, and reaches full opacity (T < 1%) during charge for *λ* < 515 nm. To test the stability of the smart window once charged, the optical features of the device were monitored by including at the end of the charging step a gradually increasing resting time under open circuit voltage (OCV) (Figure S6, Supporting Information). The resulting transmittance (at 633 nm) of the charged device shows no evolution under OCV after 1 h and less than 1% increase after 8 h, while the capacity recovered from the subsequent discharge remains very high (i.e., 98.7%). Note that the bistability of the device is intrinsic to the two redox couple exploited at the negative and positive electrode. In the discharged/transparent state, Mn^2+^ cannot react spontaneously with either Zn^2+^ or Zn because of their respective standard potentials (1.23 V and −1.18 V for Mn^IV^O_2_/Mn^2+^ and Mn^2+^/Mn^0^, respectively compared to −0.76 V for Zn^2+^/Zn^0^).^[^ ^41^ ^]^ In the charged/colored state, the reaction between MnO_2_ and Zn is thermodynamically favored, but both solid reactants are physically separated, each being deposited on a different electrode. Accordingly, the device is stable in the charged/colored state, without the need of a separator, and the spontaneous oxidation of Zn by MnO_2_ only occurs once both electrodes are connected to a resistance (or a potentiostat).

When the device is cycled over the same areal capacity (i.e., 0.2 C cm^−2^), the Coulombic efficiency and optical contrast retention remain stable at very high values over almost 200 cycles (Figure 2E, average CE of 99.5 ± 0.3%; optical contrast retention > 99.4%). At the same time, charge and discharge potentials tend to decrease slightly during cycling (by ca. ‐60 mV over 200 cycles), while voltage hysteresis remains almost constant (Figure 2D). Consequently, the energy efficiency maintains a high value of 92%, even after 180 cycles. As the cycle continues, we observe a degradation in CE, optical contrast retention, and voltage hysteresis (Figure 2D,E). We also notice an accumulation of MnO_2_ at the edges of the window (see Figure S7, Supporting Information). After cycling, the device was disassembled and the GLAD‐ITO electrode rinsed and rejuvenated by simply soaking in an aqueous solution containing 0.3% hydrogen peroxide (30%) and 0.4 m acetic acid.

To complete this study and assess the color switching times, we also carried out potential step chronoamperometric experiments. To achieved this, the smart window was subjected to the following potential step cycle with a first potential stepped at 1.8 V until a load of 0.2 C cm^−2^ was reached, followed then by a second potential stepped at 1.2 V for 400 s. The corresponding data are shown in Figure S8 (Supporting Information). Under these experimental conditions, the overall CE of the first cycle is 98.7%. The transmittance spectrum of the device after one complete cycle overlaps with that of the initial device, thereby confirming no significant accumulation of MnO_2_, allowing for further cycling. Regarding the color switching times, we note that charge/coloration of the device is slow, with the associated current density remaining < 5 mA cm^−2^ during the ≈100 s required to reach full charge. *On contrario*, the discharge/bleaching process is faster, with the device recovering full transparency over ≈30 s. These color switching times are much shorter than the ≈667 s associated to the galvanostatic experiments performed at 0.3 mA cm^−2^. That a faster process is observed with the chronoamperometric technique is consistent with the fact that significant overvoltages are applied during charges and discharges. Note that the faster color switching however comes at the expense of the energy efficiency of the cycle, which is significantly lower in the case of the chronoamperometric cycle (66%) compared to the galvanostatic one (92%). This is simply the consequence of the much larger voltage hysteresis between charging/coloring and discharging/bleaching steps of the former (i.e., 0.6 V compared with 0.11 V). Also, the faster switching time of the bleaching process compared to the coloring one is in line with the higher overpotential applied during the discharging/bleaching step (i.e., 0.4 V) compared to the charging/coloring step (i.e., 0.2 V). A further reduction in switching times could be achieved by increasing the overpotential values, the 0.6 V potential step being much lower than what is generally used in the literature to reversibly switch from the bleached to the colored state (typically 1.4 V for reversible metal electrodeposition).^[^ ^30^, ^42^ ^]^ However, further increase of the charging/coloring potential was not performed to avoid water oxidation at the positive electrode, an irreversible electrochemical reaction reported to compete with MnO_2_ electrodeposition at high potential values, thereby decreasing the Coulombic efficiency of reversible MnO_2_ electrodeposition.^[^ ^43^, ^44^ ^]^ Also, since the energy efficiency of the chronoamperometric cycling is already low, we did not further decrease the discharging/bleaching potential, which would otherwise decrease the cycle's energy efficiency and thus the overall charge storage performance of the device. In conclusion, although it enables fast color switching, the chronoamperometric technique is not well‐suited to ESSWs, which require a tradeoff between color switching time and energy efficiency.

**Table** **1** summarizes the charge storage performances of the Zn/ITO_1.6_ smart window compared with the other aqueous Zn‐based ESSWs devices so far reported in the literature. The present Zn/ITO_1.6_ smart window shows the highest discharge voltage, as well as the highest value of retrieved energy density (i.e., 860 mW h m^−2^ at a high power density of 4.65 W m^−2^) so far reported for an aqueous Zn‐based ESSW. Due to the high voltage discharge and the high stored energy density, a single Zn/ITO_1.6_ smart window charged to 0.2 C cm^−2^ can successfully power a 1.7 V red LED for over 10 min (see Figure S9, Supporting Information).

**Table 1:** Charge storage performances of Zn‐based ESSWs devices, obtained either from chronoamperometric (CA) or chronopotentiometric (CP) cycling experiments. Rate, capacity and average discharge voltage are related to CP (i.e., galvanostatic) experiments.

Cathode material	Charge rate [mA cm^−2^]	Capacity [mA h m^−2^]	Average discharge voltage [V]	Retrieved energy density [mW h m^−2^]	Energy efficiency [%]	Ref.
Cathodic electrochromic cathodes (self‐charging devices)						
WO_3_	0.5	185.6	≈0.2	n.r.	50 (CP)	[16]
MoTiWO_3_		260		n.r.	n.r.	[17]
V_3_O_7_	0.021^a)^	310^a)^	≈0.75	32.6 (CA)	16 (CA)	[20]
Fe‐CP	0.02	14.5	≈1.7	n.r.	n.r.	[21]
Li_4_Ti_5_O_12_	0.5	307.7	< 0.2	53 (CP)	50.3 (CP)	[22]
Nb_18_W_16_O_93_	0.25	106.7	≈0.3	n.r.	n.r.	[23]
PANI	0.026	163.6	≈0.9	113.6 (CP)	n.r.	[27]
Anodic electrochromic cathodes (self‐discharging devices)						
PB	0.007	78.9	1.24	96 (CP)	95 (CP)	[19]
PB				48 (CA)	36 (CA)	[18]
PB	0.25			50 (CP)	78 (CA charge /CP discharge)	[28]
PB	0.02	77.1	≈1.2	n.r.	n.r.	[25]
MnO_2_‐PANI	0.072	1270	≈0.9	n.r.	n.r.	[24]
MnO_2_	0.3	555	1.56	860 (CP)	93.4 (CP)	This work
		555		657 (CA)	66 (CA)	This work

^a)^ Values estimated from the data in ref. [20], with a maximal capacity of 145 mA h g^−1^, considering 0.6 mg of active material deposited on a 8 × 35 mm^2^ electrode surface.

n.r. = not reported

The long term cyclability of the Zn/ITO smart window is currently limited by the gradual accumulation of MnO_2_ at the edges of the GLAD‐ITO electrode. Since GLAD‐ITO electrodes were already previously demonstrated to enable reversible electrodeposition of MnO_2_ over 1000 cycles in a large cell assembly,^[^ ^45^ ^]^ we can assume here that the observed degradation is probably linked to constraints related to the smart window architecture. Indeed, the use of a zinc metal frame leads to some drawbacks. The first one is related to the inhomogeneous electric field lines during cycling. Even if the MnO_2_ deposit appears nicely homogeneous at a macroscopic scale, we cannot rule out that it is indeed imperfectly homogeneous at the microscale, leading to inhomogeneous accumulation of MnO_2_ close to the zinc frame with long term cycling. If true, this effect could be exacerbated for larger area devices. The second one is that competitive hydrogen evolution occurs at the zinc anode during charge, leading to bubbles formation during cycling. Over a long cycling period, this can lead to an increase in pH, despite the use of a buffered electrolyte, which may explain the gradual shift in the charge/discharge potentials observed over prolonged cycling (Figure 2D). One can thus assume that a smart window based on a face‐to‐face assembly of two transparent conductive windows, each independently colored by electrodeposition of Zn or MnO_2_ upon charge, would alleviate these problems and thus improve cyclability. At the same time, the color‐neutrality and opacity of the device in its charged state should be improved.

**Figure 3:**
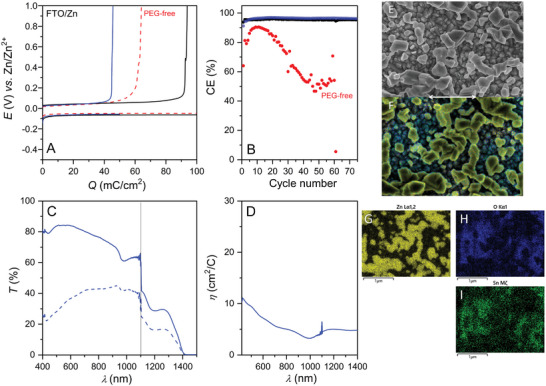
Galvanostatic cycling of a FTO/Zn device filled with (black, blue) the optimized PEG‐containing electrolyte or (red) the PEG‐free electrolyte. The charge was initially fixed at (blue) 0.05 or (red, black) 0.1 C cm^−2^, and the rate was (red) 0.3 or (black, blue) 1 mA cm^−2^. A) First galvanostatic cycles of zinc electroplating/electrostripping. B) Coulombic efficiency. C) Transmittance spectra of the (solid line) initial and (dashed line) Zn‐deposited device. D) Coloration efficiency as a function of wavelength. E) SEM image and (F‐I) EDS mapping of the FTO electrode after Zn electroplating to a charge of 0.1 C cm^−2^ at 1 mA cm^−2^ in the optimized PEG‐containing electrolyte. In (F‐I), the same region is represented in each panel, and the elemental color code is as follows: (yellow) Zn, (blue) O, (cyan) F, and (green) Sn. Scale bar is equivalent to 1 µm.

However, achieving reversible zinc electrodeposition at a transparent conductive oxide (TCO) electrode represents a major challenge. Indeed, TCOs are characterized by a low surface energy and a poor wettability, which translates into a high nucleation energy barrier for metal electroplating. Consequently, sparse and irregular grains grow on a small number of nucleation sites, causing poor surface adhesion and inhomogeneous dendritic deposits.^[^ ^46^ ^]^ To date, only one study reports on RZE at a transparent ITO‐coated glass substrate,^[^ ^31^ ^]^ combined to a Zn mesh counter electrode in a dynamic window assembly. The cyclability of the windows was found to be limited by the co‐precipitation of ZnO and Zn(OH)_2_, leading to a continuous decrease of the optical contrast.^[^ ^31^ ^]^ To overcome this limitation, we decided here to take advantage of a mild aqueous buffered electrolyte in order to locally stabilize the pH and avoid the precipitation of such zinc hydroxides. We also selected FTO as transparent electrode for RZE because of its improved electrochemical stability at negative potentials as compared to ITO (this later being more prone to degradation by proton‐coupled electrochemical reduction in aqueous electrolytes).^[^ ^47^, ^48^ ^]^ Accordingly, we constructed a symmetrical FTO/Zn smart window assembly, combining an FTO working electrode with a perforated zinc grid counter‐electrode, silicone frame separators and a sealing glass window (see scheme in Figure S4, Supporting Information). In a first attempt, this assembly was galvanostatically cycled under conditions close to those previously used with Zn/ITO smart windows, including both the cycling parameters (load = 0.1 C cm^−2^ – equivalent to a Zn mass load of ≈34 µg cm^−2^ – and rate = 1 mA cm^−2^) and electrolyte composition (1 m acetate buffer of pH 4.5 along with 0.2 m ZnCl_2 _). The electroplating potential curve was characterized by a short potential overshoot up to −0.08 V, typical of a nucleation and growth process, followed by potential stabilization at −0.05 V (red dashed curve in **Figure** **3**A). Concomitantly, the transmittance of the device continuously decreases (Figure S10, Supporting Information). The subsequent electrostripping step is also characterized by a highly stable potential, but with a limited discharge capacity that leads to a low CE (65%). The CE also remains low upon cycling, with an average value of 72.8% over 50 cycles (red dots in Figure 3B) and a high standard deviation of 14.7%, while the transparency retention of the device in the bleached state was also observed to progressively decrease (Figure S10, Supporting Information).

In order to improve the efficiency, reversibility, and cyclability of RZE at the FTO electrode, the cycling parameters as well as chemical composition of the electrolyte have been thoroughly investigated and optimized,^[^ ^49^ ^]^ and a detailed presentation of this work will be the subject of a forthcoming paper. The best performances of the FTO/Zn smart window were obtained using the following optimized electrolyte: 1 m acetate buffer, containing 0.5 m Zn(Ac)_2_ and 10% vol. PEG‐200 (pH 5.0). This latter soluble additive has been reported to suppress dendrite growth during zinc electrodeposition^[^ ^50^ ^]^ and to improve the cyclability and reversibility of Zn anodes.^[^ ^51^ ^]^ In this optimized electrolyte, a much better CE is obtained during the first cycle (> 90%), and we observed further increases with the cycling until it stabilizes at a value > 95% for over 75 cycles (see Figure 3B, with average CE values of 96.4 ± 0.7% and 95.5 ± 0.3% for a charge set at 0.05 and 0.1 C cm^−2^, respectively). This allows the device to retain good transparency in the bleached state upon cycling (Figure S10, Supporting Information). The coloration efficiency was estimated to range from 3 to 11 cm^2^ C^−1^ for a load set to 50 mC cm^−2^, depending on the wavelength considered (Figure 3D). These values are below the theoretical value of 59 cm^2^ C^−1^ calculated in the absence of reflection,^[^ ^30^ ^]^ and the values of ≈20 cm^2^ C^−1^ expected for a dense film of Zn with thickness over 200 Å.^[^ ^52^ ^]^ We assume that the lower values reported here arise from a non‐compact Zn deposit with decreased light‐blocking properties because of the embedded voids.

A further analysis of the zinc deposit was done by *ex‐situ* SEM analysis of the FTO electrode charged to 0.1 C cm^−2^ (3rd cycle). The SEM images (Figure 3E,F; Figure S11, Supporting Information) show highly homogeneous deposition of submicrometer‐sized Zn metal particles on the FTO electrode, itself composed of tightly‐packed grains roughly 100–300 nm in diameter. The Zn deposit is free of dendritic growths and filament‐like structures, and mostly constituted of layer‐like particles. The elemental EDS mapping confirmed the pure zinc composition of the particles with concomitant depletion of the surface in both oxygen and tin (Figure 3G‐H). The morphology of the Zn electrodeposit on FTO is found to be quite similar to what has been recently reported for the galvanostatic deposition of Zn on Cu from a 1 m Zn(Ac)_2_ aqueous electrolyte, also resulting in non‐compact deposits of layer‐like particles for charges < 1 mA h cm^−2^ (eq. to 3.6 C cm^−2^).^[^ ^53^ ^]^

**Figure 4:**
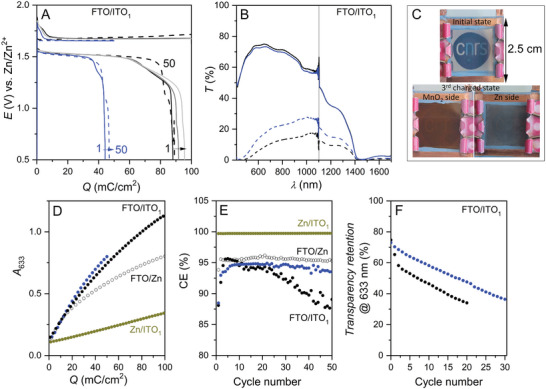
Galvanostatic cycling of FTO/ITO_1_ devices at 1 mA cm^−2^ for a load set at (black) 0.1 or (blue) 0.05 C cm^−2^. The electrolyte is 1 m acetate buffer along with 0.4 m Zn(Ac)_2_, 0.1 m Mn(Ac)_2_, and 10% vol. PEG‐200. A) Galvanostatic charge/discharge profiles of the device charged to 0.1 C cm^−2^ for the (solid black) 1st, (grey) 2nd, (light grey) 5th, and (dashed black) 50th cycles, and of the device charged to 0.05 C cm^−2^ for the (solid blue) 1st and (dashed blue) 50th cycles. B) Transmittance spectra of the (solid lines) corresponding as‐fabricated, unloaded devices and (dashed lines) devices after 1st complete charging process. C) Photographs of the FTO/ITO_1_ smart window at different states of charge. D) Absorbance monitored at 633 nm during the first charge. E) Coulombic efficiency over 50 cycles. F) Retention of transparency of the discharged state upon cycling, monitored at 633 nm. Overlaid in (C) and (D) are the data obtained upon cycling the single‐tinting (dark yellow) Zn/ITO_1_ or (empty dots) FTO/Zn devices for a charge set at 0.1 C cm^−2^ and a rate of 1 mA cm^−2^.

Based on these encouraging results, we then built a proof‐of‐concept FTO/ITO_1_ smart window demonstrating a dual‐tinting ESSW through the exploitation of complementary reversible electrodeposition reactions at symmetrically positioned transparent electrodes (Figure S4, Supporting Information). For this assembly, we selected a 1 µm‐thick GLAD‐ITO electrode and limited the load to 0.05 or 0.1 C cm^−2^. We first confirmed the performance of reversible MnO_2_ electrodeposition at the ITO_1_ electrode under the experimental conditions optimized for RZE at the FTO electrode. This was achieved using a Zn/ITO_1_ smart window assembly, filled with an aqueous electrolyte made of 1 m acetate buffer along with 0.4 m Zn(Ac)_2_, 0.1 m Mn(Ac)_2_ and 10% vol. PEG‐200, and charged to 0.1 C cm^−2^ at 1 mA cm^−2^. The resulting data shown in Figure S12 (Supporting Information) demonstrates the excellent efficiency and reversibility of MnO_2_ electrodeposition under these conditions, as attested by the high and stable average CE (99.7 ± 0.01%) over 65 cycles, as well as stable optical retention. Having demonstrated the compatibility of the optimized electrolyte with both electrodeposition reactions, we next investigated the FTO/ITO_1_ smart window under identical experimental conditions, by setting the charge to 0.1 or 0.05 C cm^−2^. The collected data are gathered in **Figure** **4**.

During the charging process, nucleation at both electrodes is accompanied by an initial burst of potential up to 1.8 V, which then rapidly decreases to reach a highly stable value of 1.65 V (Figure 4A). Concomitantly, the transmittance of the device decreases below 20% over the 420–1700 nm wavelength range for the device charged up to 0.1 C cm^−2^ (Figure 4B). The color of the device in the charged state is highly homogeneous on both sides, with the anode side presenting a gray coloration and the cathode side showing a brown coloration (Figure 4C).

Evolution of the absorbance at 633 nm during the first charge (Figure 4D) shows a linear relationship at the beginning (*Q* < 30 mC cm^−2^), enabling calculation of a coloration efficiency of 17 cm^2^ C^−1^. Note that the variation in transmittance/absorbance is much more marked for the dual‐tinting FTO/ITO_1_ device than for the single tinting devices, whether for the Zn/ITO_1_ (relying on reversible MnO_2_ electrodeposition at the ITO cathode, see dark yellow dots in Figure 4D) or the FTO/Zn (relying on RZE at the FTO anode, see empty dots in Figure 4D) device. This clearly demonstrates the benefits of combining the two electrodeposition processes to increase both the optical contrast and wavelength range for opacification. During discharge, the potential curve is centered on 1.51 V, leading to an overall hysteresis of 0.15 V and a Coulombic efficiency of ≈88%, whatever the charge. Upon cycling, the charge/discharge potentials remain quite stable, while CE increases. CE reaches a maximal value of ≈95% after a few cycles (Figure 4E), and then remains quite stable over 50 cycles when the charge is limited to 0.05 C cm^−2^, but decreases rapidly if the charge is higher. This CE value is similar to that of RZE on FTO (see empty dots in Figure 4E), but much lower than that of the reversible MnO_2_ electrodeposition on ITO_1_ (see dark yellow dots in Figure 4E). As a result, MnO_2_ accumulate at the cathode with each cycle, which explains the progressive loss of device transparency in the discharged state upon cycling (Figure 4F). This decrease is more pronounced as the charge capacity is increased (compare black and blue dots in Figure 4F), simply because the amount of MnO_2_ accumulated at the cathode per cycle is higher.

To complete this study, we also submitted a Zn/ITO_1_ device to chronoamperometric cycles following the initial galvanostatic cycle, with the charge being set at 0.1 C cm^−2^. The corresponding data are shown in Figure S13 (Supporting Information). Again, the color switching time is strongly dependent on the electrochemical parameters selected, with the color switching time decreasing here from 100 s in the galvanostatic experiments to ≈30 s in the potential step chronoamperometry, but at the expense of the energy efficiency. Also, gradual MnO_2_ accumulation at the cathode was observed upon cycling, and this independently of the cycling procedure.

To further demonstrate the benefits of the face‐to‐face assembly for obtaining perfectly homogeneous deposits at the two electrodes, we also assembled and tested a larger dual‐tinting FTO/ITO_1_ device. It was made using 5×5 cm^2^ electrodes, presenting an electroactive surface area of ≈18 cm^2^ (i.e., 5‐times larger than the smaller devices). It was filled with the optimized electrolyte and cycled under the usual conditions. The photographs taken throughout the first cycle (Figure S14, Supporting Information) demonstrate a perfectly homogeneous deposit at both the anode (Zn) and cathode (MnO_2_) sides. The Coulombic efficiency of the first cycle (i.e., 88.5%) is also identical to that previously obtained with the smaller device, while the voltage hysteresis is slightly increased to 0.23 V. These data demonstrate the successful upscaling of the device and the suitability of the galvanostatic cycling mode for achieving homogeneous deposition at moderate current densities, thereby mitigating the voltage drop across the transparent conductive electrodes.^[^ ^54^ ^]^ The light yellow coloration of the device at the end of the first cycle attests of the accumulation of MnO_2_ at the cathode during cycling, in line with what we have also obtained for the smaller device and which here again results from the moderate CE value.

**Figure 5:**
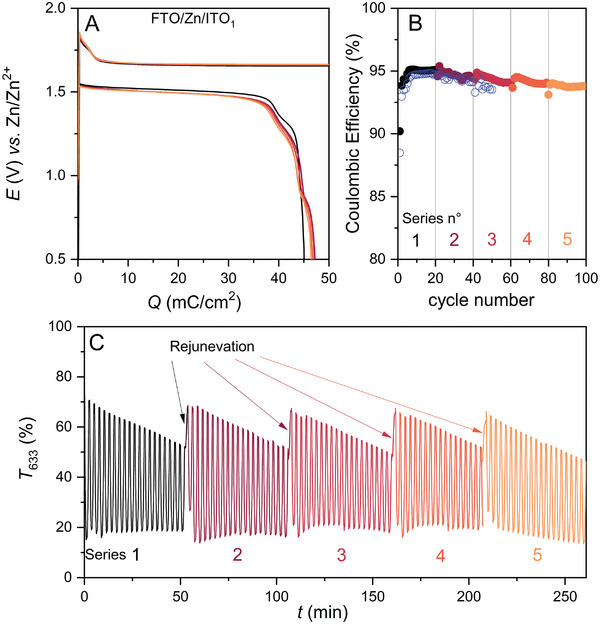
Galvanostatic cycling of a FTO/Zn/ITO_1_ device at 1 mA cm^−2^ for a series of 100 cycles, with a load of 0.05 C cm^−2^, and with a specific galvanostatic cathode discharge step incorporated every 20th cycle. A, from black to orange) Charge/discharge potential curves recorded following each rejuvenation process after a series of 20 cycles. B) Coulombic efficiency over the 5 successive series of 20 cycles together with (blue dots) values obtained for the continuous cycling of the FTO/ITO_1_ device without rejuvenation. C) Transmittance continuously monitored at 633 nm during cycling, including rejunevation steps.

It is worth noting that color accumulation upon continuous cycling was also reported by Kang, Gao and coll. in a recent work on a smart window assembly based on the complementary reversible electrodeposition of MnO_2_ and Cu on planar FTO electrodes.^[^ ^55^ ^]^ In that study, the issue could be mitigated upon addition of FeCl_3_, a soluble redox mediator allowing to trigger MnO_2_ electrodissolution, although at a very low voltage detrimental to the overall energy efficiency of the device. In the present case, coloration accumulation at the positive MnO_2_ electrode is not induced by accumulation of a redox‐inactive MnO_2_ fraction, but from the lower efficiency of RZE at the counter‐electrode. In order to improve the cyclability of the dual‐tinting device, it is thus mandatory to increase the Coulombic efficiency of RZE at the transparent anode beyond what is achieved here. Preliminary experiments performed with a slight modification of the ratios of the two multivalent cations (while keeping the ionic strength constant) show that the device's efficiency is quite sensitive to the chemical composition of the electrolyte (Figure S15, Supporting Information). Further studies are therefore required, and work is in progress to improve the system. However, even if we can hope to improve the Coulombic efficiency of the device by adjusting the electrolyte composition and cycling conditions, it will still be difficult to achieve perfect balance in a configuration that implements simultaneous reversible electrodeposition processes at each window electrode. This is clearly an intrinsic issue of this kind of ESSW device. Accordingly, suitable rejuvenation strategies need to be considered. In the case of MnO_2_ accumulation at the cathode, rejuvenation strategies could rely on flushing the device with a slightly acidic electrolyte containing hydrogen peroxide, but this is likely difficult to implement in practice. Another strategy relies on the use of an additional sacrificial zinc anode for *in‐situ* cathode rejuvenation. As a proof‐of‐concept, we constructed a 3‐electrode FTO/Zn/ITO_1_ smart window, incorporating a zinc frame as a sacrificial anode (see scheme in Figure S16, Supporting Information). The following sequential protocol was used: (*i*) the ITO_1_ cathode was first coupled with the FTO anode (i.e., equivalent to a FTO/ITO_1_ smart window) and submitted to a series of 20 galvanostatic charge/discharge cycles with a charging rate fixed at 1 mA cm^−2^ and a load set to 0.05 C cm^−2^; (*ii*) the FTO anode was interchanged with the Zn frame anode (i.e., equivalent to a Zn/ITO_1_ smart window) and the device submitted to two successive galvanostatic discharge rejuvenation steps, first at 1 mA cm^−2^ followed then by a second at 0.3 mA cm^−2^. This two‐step process was repeated 5 times, for a total of 100 charge/discharge cycles. The results are gathered in **Figure** **5**.

The results obtained for the first series of 20 cycles are totally superimposable on those obtained with the previous FTO/ITO_1_ device (as attested by the near‐overlap of their Coulombic efficiencies shown in Figure 5B, stabilizing at ≈95% after a few initial cycles). During this cycling, the transparency of the device in the discharged state decreases continuously, most notably the transmittance at 633 nm (Figure 5C). During the subsequent *in‐situ* discharge of the cathode versus the sacrificial zinc anode, a charge of *ca*. 50 mC cm^−2^ is recovered (Figure S17, Supporting Information). This amount of charge is in full agreement with the net charge lost due to MnO_2_ accumulation over 20 cycles with an average CE of 94.7 ± 1.1% (i.e., ≈5 mC cm^−2^ per cycle for 20 cycles). This indicates that all the accumulated MnO_2_ can be electrodissolved in this step, enabling the device to recover high transmittance in its discharged state (Figure 5C). The results obtained for the second (and subsequent) series of 20 cycles are all very similar to those of the first series. The charge/discharge potentials of the first galvanostatic cycles recorded for each series of 20 cycles almost overlap (Figure 5A), including the potential burst associated with the nucleation step at the early stage of charging, which confirms that each series of cycles starts from nearly the initial state. The average CE over the 20 cycles remains high and very stable (94.7 ± 0.25%, 94.4 ± 0.25%, 94.2 ± 0.22%, and 93.8 ± 0.19% for series 2 to 5), as does the EE (≈82.5%), leading for each series to very similar values of transmittance decrease at 633 nm and recovered capacity during in situ cathodic discharge. Overall, these results clearly demonstrate the benefits of the rejuvenation strategy to increase the cyclability of the device. *En passant*, it is also worth noting that the charge and discharge potentials remain very stable during cycling, in contrast to what is reported for the Zn/ITO_1.6_ device (see Figure 2D). This seems to confirm that the absence of a large excess of zinc limits the hydrogen evolution during cycling.

## 3 Conclusion

The work presented here constitutes a comprehensive study of energy storage smart windows which, for the first time, exploit complementary reversible electrodeposition reactions at both the anode and cathode. Different architectures were characterized, all assembled in a transparent discharged state and operating with a mild aqueous electrolyte containing the electroactive Zn^2+^ and Mn^2+^ multivalent cations. Here, we have successfully transposed the chemistry of rechargeable Zn/MnO_2_ batteries in mild aqueous electrolytes to bifunctional smart window devices. This approach significantly improves the charge storage performances compared to other Zn‐based ESSWs so far reported in the literature. Furthermore, we demonstrated for the first time that reversible zinc electrodeposition at a transparent FTO electrode can be achieved with a remarkably good efficiency, allowing for the development of an advanced dual‐tinting ESSW device. The advantages of such device have been clearly established, notably in terms of coloration over a wide range of wavelength, as well the constraints and limitations, particularly those associated with cyclability, leading us to propose an effective procedure for device rejuvenation. Finally, although the cyclability still needs to be improved, the proof‐of‐concept provided here paves the way for the design of new multifunctional electrochromic devices for energy‐saving applications, whose scope extends beyond smart windows to notably include smart batteries and supercapacitors state‐of‐charge sensors.

## 4 Experimental Section

### Chemicals and Materials

All chemicals were bought from commercial suppliers and utilized in their original states without any additional purification steps. ZnCl_2_, MnCl_2_ (≥99%), Zn(CH_3_COO)_2_,2H_2_O (>99.5%), Mn(CH_3_COO)_2_,4H_2_O (≥99.5%), polyethylene glycol (PEG)−200, CH_3_COOH (≥99.5%), CH_3_COOK (≥99%), and ethanol were purchased from Sigma Aldrich. Glycerol (≥99%) was purchased from Thermo Fisher Scientific. Dichloromethane (100%) and acetone (100%) were purchased from VWR chemicals. All aqueous solutions were prepared using highly purified Milli‐Q water (18.2 MΩ cm). Zinc (99.5%) as 0.25 mm thick metallic sheets were purchased from Goodfellow. Glass‐ITO substrates (8‐12 Ω/□) were purchased from Delta Technologies. Glass‐FTO electrodes (7‐10 Ω/□) were purchased from Solems. Conductive copper tape (3 m) and silicone rubber sheets (1.5 mm thick) were purchased from RS Components (France).

### GLAD Electrodes

The mesoporous ITO film was deposited by glancing angle deposition (GLAD) on 2.5 × 2.5 cm^2^ (alternatively 5 × 5 cm^2^) commercially‐sourced glass/ITO substrates. The substrates were cleaned by hand in soapy water, successively sonicated in water and isopropanol, then blown dry in an N_2_ stream. The substrates were then mounted on an aluminum deposition plate using Kapton tape to mask ≈2.5 mm wide areas around each edge. Accordingly, ITO film was deposited within the ca. 2 × 2 cm^2^ (alternatively ca. 4.5 × 4.5 cm^2^) interior, while the perimeter remained uncoated. The deposition plate was loaded into a GLAD‐equipped and feedback‐controlled electron‐beam evaporation system (Kurt J. Lesker), and ITO pellets (Materion, 91:9 In_2_O_3_/SnO_2_, 99.99%) were placed in a graphite crucible liner and inserted into the e‐beam source. Prior to deposition, the vacuum chamber was evacuated to <5 × 10^−5^ Pa, and during deposition, a 7.5 kV electron accelerating voltage was applied while the e‐beam current (≈40–60 mA) and position were continuously adjusted to maintain a ≈0.4 nm s^−1^ deposition rate as measured by quartz crystal microbalance. Post‐like structures were formed by positioning the deposition plate at a fixed 85° angle with respect to the direction of ITO vapor incidence, and rotating the plate at a rate of 1 revolution per 10 nm of deposited film. Following deposition, the GLAD‐ITO electrodes were subjected to a two‐stage anneal process to improve both transparency and electrical conductivity. First, the electrodes were annealed in an air environment at 500 °C for 1.5 h, followed by annealing in forming gas (5% H_2_/Ar) at 300 °C for 1 h.

### Film Characterization

SEM images of the bare and MnO_2_‐modified GLAD‐ITO electrodes were recorded using a Hitachi S‐4800 cold field emission SEM, operating at a 5 kV accelerating voltage. SEM and EDS images of the bare and Zn‐modified FTO electrodes were recorded using a Zeiss Gemini SEM 360 (resolution: 0.7 nm/15 kV‐1.2 nm/1 kV).

### Aqueous Electrolytes

1 m Acetate buffer + 0.1 m MnCl_2_ + 0.1 m ZnCl_2_: 1 m acetate buffer was prepared in a 20 mL volumetric flask from 1.25 g of potassium acetate and 415 µL of acetic acid, then the flask was filled with water to a total volume of 20 mL. An additional 395 mg of MnCl_2_ and 272 mg of ZnCl_2_ was then added. The pH of the electrolyte was measured and found to be 4.8. 1 m Acetate buffer + 0.4 m Zn(CH_3_COO)_2_ + 0.1 m Mn(CH_3_COO)_2_ + 10% PEG‐200: in a 20 mL volumetric flask, 1.25 g of potassium acetate and 415 µL of acetic acid were introduced and dissolved in a small amount of water. Next, 1.76 g of Zn(CH_3_COO)_2_,2H_2_O and 0.49 g of Mn(CH_3_COO)_2_,4H_2_O were introduced and dissolved by adding a small volume of water. Finally, 2 mL of PEG‐200 were added, and the flask was shaken vigorously before filling to 20 mL with water. The electrolyte was then transferred to a small glass vial and stirred for 2 hrs at room temperature. The pH of the electrolyte was measured and found to be 5.1. 1 m Acetate buffer + 0.5 m Zn(CH_3_COO)_2_ + 10% PEG‐200: in a 20 mL volumetric flask, 1.25 g of potassium acetate and 415 µL of acetic acid were introduced and dissolved in a small amount of water. Next, 2.20 g of Zn(CH_3_COO)_2_,2H_2_O was introduced and dissolved with the minimal amount of water. Finally, 2 mL of PEG‐200 were added, and the flask was shaken vigorously before filling to 20 mL with water. The electrolyte was then transferred to a small glass vial and stirred for 2 hrs at room temperature. The pH of the electrolyte was measured and found to be 5.0.

### Dynamic Window Assemblies

The GLAD‐ITO electrodes were cleaned by successive immersion in chloroform, acetone, and ethanol at 50 °C for 30 min each. The commercial FTO electrodes were cleaned by sonication in successive baths of dichloromethane, acetone, and ethanol (5 min each). To improve electrical connectivity, a conductive copper tape was applied to the outer edges of all transparent electrodes, and the tape was subsequently covered with an insulating layer to ensure no Cu ions contaminate the electrolyte. All windows were assembled in their transparent state, and sealed with clips. *Single‐tinting Zn/ITO assembly*: The 2.5 × 2.5 cm^2^ Zn/GLAD‐ITO smart window was assembled by combining a GLAD‐ITO electrode with a silicone polymer frame as first separator, a Zn metal frame as the counter electrode, a second silicone polymer frame as second separator, and a microscope slide as back glass window. Once assembled, the device was filled with ≈1.2 mL of a Zn^2+^/Mn^2+^‐based aqueous buffered electrolyte. *Symmetric FTO/Zn assembly*: The 2.5 × 2.5 cm^2^ Zn/Zn smart window was assembled by combining an FTO electrode with a silicone polymer frame as first separator, a Zn metal frame as the counter electrode, a second polymer frame as second separator, and a microscope slide as back glass window. Once assembled, the device was filled with ≈1.2 mL of a Zn^2+^‐based aqueous buffered electrolyte. *Dual‐tinting Zn/MnO_2_ assembly*: The 2.5 × 2.5 cm^2^ (alternatively 5 × 5 cm^2^) FTO/GLAD‐ITO smart window was assembled by combining a GLAD‐ITO electrode, one frame of silicone polymer, and a FTO counter electrode. Once assembled, the device was filled with a volume <1 mL of a Zn^2+^/Mn^2+^ based aqueous buffered electrolyte including the PEG‐200 additive. *FTO/Zn/MnO_2_ assembly with sacrificial zinc anode*: The 2.5 × c2.5 cm^2^ FTO/Zn/GLAD‐ITO smart window was assembled by combining a GLAD‐ITO electrode, one frame of silicone polymer, a zinc frame, a second frame of silicone and an FTO counter electrode. Once assembled, the device was filled with a volume of ≈1.2 mL of a Zn^2+^/Mn^2+^ based aqueous buffered electrolyte including the PEG‐200 additive.

### Spectroelectrochemistry

All electrochemical galvanostatic experiments were carried out using a VSP BioLogic potentiostat controlled by EC‐Lab 11.3 software. The charge/discharge current densities were fixed to ± 0.3 or ± 1 mA cm^−2^. For the Zn/ITO and FTO/ITO assemblies, the amount of charges (or load, or areal capacity) passed during charging was set to a fixed value as specified in the text, while a cut‐off potential of 0.5 V was applied during discharging. A resting time of 30 s was also systematically applied at the end of each charge and discharge process. For the symmetric FTO/Zn assembly, the load passed during charging was set to a fixed value as specified in the text, while a cut‐off potential of 1 V was applied during discharging. Simultaneously, the visible‐NIR transmittance spectra were recorded over 420–1700 nm using a bifurcated visible‐NIR optical fiber connected to an HR‐2000+ spectrometer (Ocean Optics) and a Flame‐NIR spectrometer (Ocean Insight), both controlled by Ocean View software. The integration times were set at 30 ms and 1 ms for the HR‐2000+ and FlameNIR spectrometers, respectively, with an average of 100 scans. Light was provided by a DH‐2000‐BAL halogen lamp (Ocean Insight). All blank spectra were recorded in air.

The optical contrast retention (OCR in %) of the device is defined as

(6) $$\mathrm{OCR}\left(\lambda \right)=\frac{\log\left(T_{discharged,i}/T_{charged,i}\right)}{\log\left(T_{initial}/T_{charged,1}\right)}\times 100$$

where *T*_*discharged*,*i*_ and *T*_*charged*,*i*_ are the transmittance of the fully discharged and charged device for cycle *i*, respectively, *T_initial_* is the transmittance of the device in its initial discharged state, and *T*_*charged*,1_ is the transmittance of the device after the first charge. All transmittance values are monitored at an identical wavelength *λ*.

The transparency retention (TR in %) of the device is defined as

(7) $$\mathrm{TR}\left(\lambda \right)=\frac{T_{discharged,i}}{T_{initial}}\times 100$$

Charge Storage performances. The Coulombic efficiency (CE in %) for each galvanostatic cycle is defined as

(8) $$CE=100\times \frac{Q_{discharge}}{Q_{charge}}$$

where *Q_discharge_* (resp. *Q_charge_*) is the amount of charge (in mC cm^−2^) passed upon discharge (resp. charge).

The energy density (in mW h m^−2^) was calculated from integration of the galvanostatic curve, corresponding thus to the following equation:

(9) E=i×∫Vdt

where *i* is the (fixed) intensity (in mA m^−2^) and *V* is the discharge voltage (in V).

The energy efficiency (EE in %) for each galvanostatic cycle is defined as

(10) $$EE=100\times \frac{E_{discharge}}{E_{charge}}$$

where *E_discharge_* (resp. *E_charge_*) is the discharge (resp. charge) energy density (mW h m^−2^) calculated from integration of the discharge (resp. charge) galvanostatic curve.

## Conflict of Interest

The authors declare no conflict of interest.

## Supporting information

Supporting Information

## Data Availability

The data that support the findings of this study are available from the corresponding author upon reasonable request.
