# Supplementary material for: Innovative Energy Storage Smart Windows Relying on Mild Aqueous Zn/MnO2 Battery Chemistry
Source: Adv Sci (Weinh). 2024 May 29;11(29):2402369. doi: 10.1002/advs.202402369 (PMC11304267; doi:10.1002/advs.202402369)
Supplement: Supplementary file 1 — Supporting Information [file ADVS-11-2402369-s001.docx]

Supplementary Information to

Innovative energy storage smart windows relying on mild aqueous Zn/MnO_2_ battery chemistry

Hamid Palamadathil Kannattil,^1^ Lluis Martinez Soria Gallo,^1^ Kenneth D. Harris,^2^ Benoît Limoges,^1^ Véronique Balland^1,*^

1. Université Paris Cité, CNRS, Laboratoire d'Electrochimie Moléculaire, F-75013 Paris, France

2. National Research Council Canada – Nanotechnology Research Centre, Edmonton, AB, Canada and Department of Mechanical Engineering, University of Alberta, Edmonton, AB, Canada

* Corresponding author : [veronique.balland@u-paris.fr](mailto:veronique.balland@u-paris.fr)


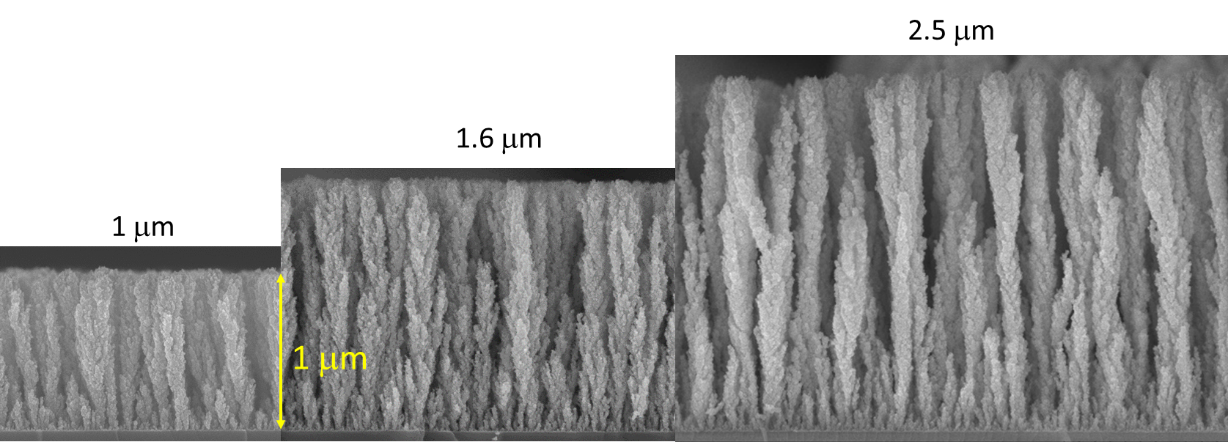


Figure S1. Cross-sectional SEM micrographs of the GLAD-ITO deposit corresponding to the ITO_1_, ITO_1.6_ and ITO_2.5_ electrodes.

Figure S2. (A) Current density measured at 0.5 V *vs*. Ag/AgCl at GLAD-ITO_x_ electrodes soaked in a 1M KCl aqueous electrolyte as a function of the scan rate, for x= (dark) 1, (red) 1.6 and (blue) 2.5 μm. Plain lines correspond to the associated linear regressions. (B) Electrical double layer capacitance of the GLAD-ITO electrodes as a function of the GLAD deposit thickness. The red line corresponds to the linear regression with intercept = 19 μF/cm^2^, slope = 236 μF/cm^2^/μm and r^2^ = 0.99127.


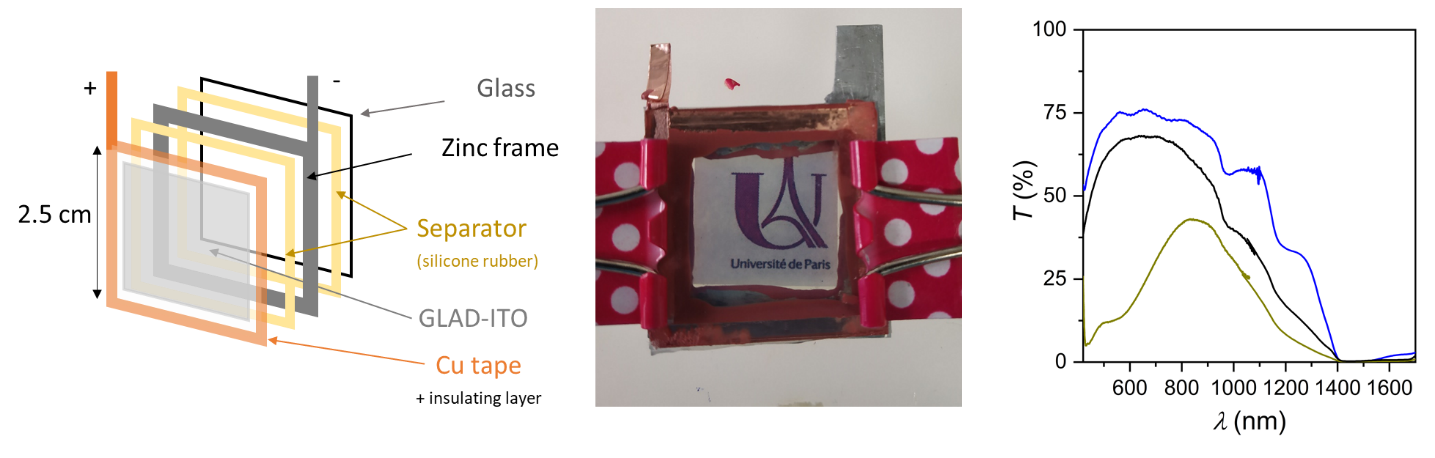


Figure S3. Device assembly, photograph and transmittance spectra of the Zn/ITO_x_ smart windows assembled using (blue) ITO_1_, (black) ITO_1.6_ and (dark yellow) ITO_2.5_ electrodes.


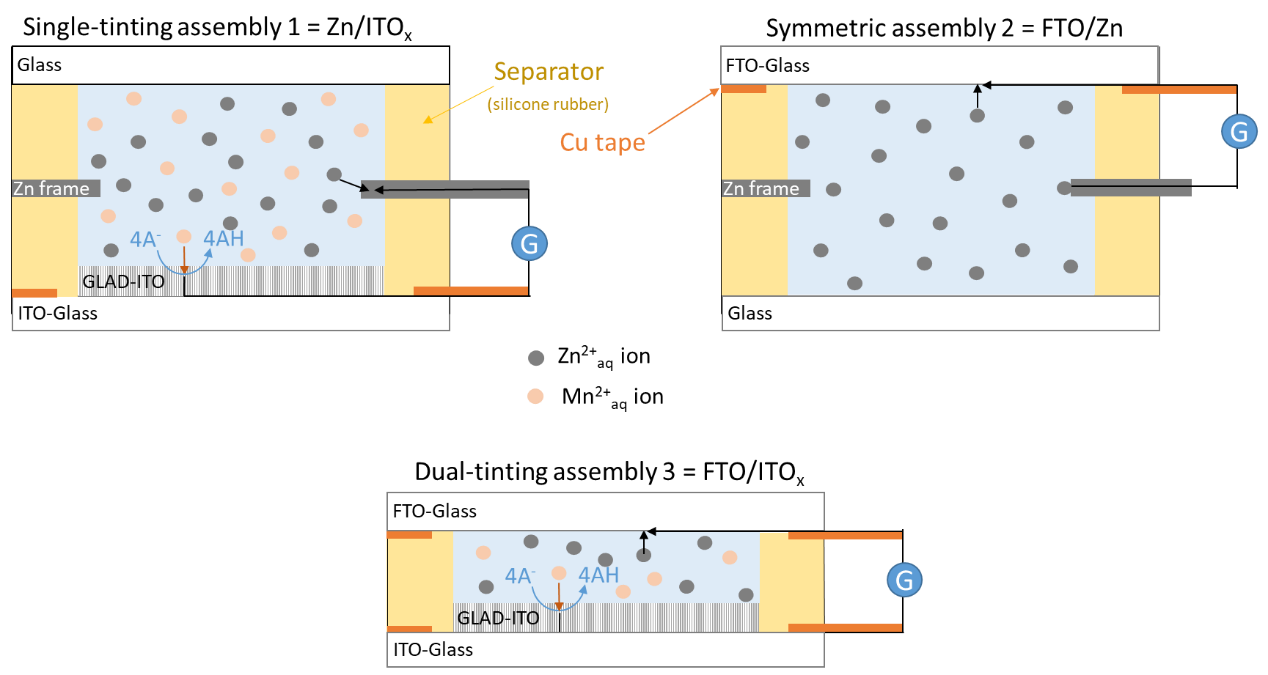


Figure S4. Coloration and/or charging principle of the various smart windows assembled in this work. AH and A^-^ refer to acetic acid and acetate. The symbol G refers to galvanostatic charge processes.

Figure S5. Galvanostatic cycling of the Zn/ITO_1.6_ device at 0.3 mA/cm^2^. The charge was initially fixed at (black data) 0.1 C/cm^2^, and stepwise increased by 0.1 C/cm^2^ every ten cycle. (A) first charge/discharge potential curves and (B) transmittance spectra are given for charges fixed at (black) 0.1 ; (blue) 0.2 ; (violet) 0.3 and (red, only in A) 0.4 C/cm^2^. The grey spectra in (B) corresponds to the transmittance of the initial device.

Figure S6. Stability test. Transmittance and voltage monitored upon cycling the Zn/ITO_1.6_ device up to a charge of 0.18 C/cm^2^ at 0.3 mA/cm^2^, either (black) continuously or (blue curves) with the inclusion of an open circuit voltage resting time at the end of the charging step. The duration of the resting time was gradually increased from (from dark to light blue) 30 min to 1 hr and then 8 hrs.


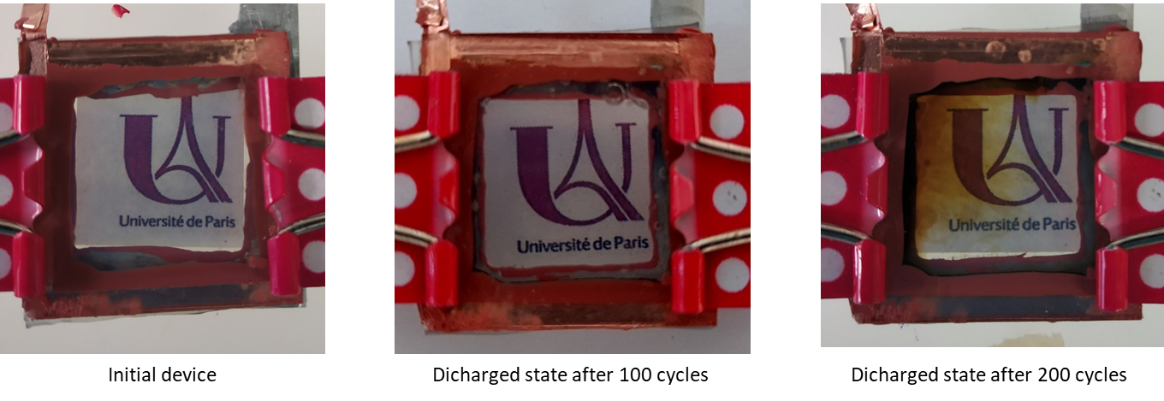


Figure S7. Photographs of the Zn/ITO_1.6_ device in (left) its initial, as-assembled bleached state, and subsequently, in its discharged state after (middle) 100 and (right) 200 charge/discharge cycles.

Figure S8. Chronoamperometric cycling of the Zn/ITO_1.6_ device, showing (A) the charge capacity over time, (B) the full transmittance spectra recorded (from black to light orange) every 25 s upon charge/coloration and (dashed line) at the end of the discharge/bleaching step, (C) the current density upon time and (D) the transmittance monitored at 633 nm over time for 4 consecutive cycles.


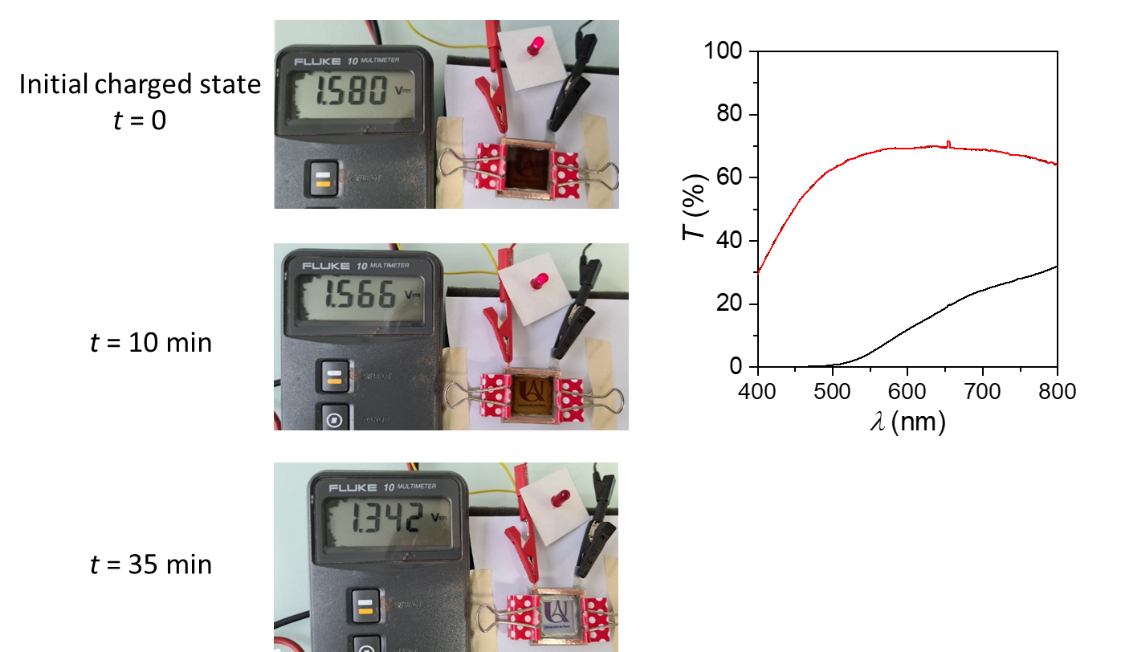


Figure S9. (Left) Photographs of the Zn/ITO_1.6_ device previously charged to 0.2 C/cm^2^ powering a 1.7 V LED during its discharge cycle. At the end of the discharge (i.e., after 35 min), the voltage delivered was too low to efficiently power the LED. (Right) Transmittance spectra of the Zn/ITO_1.6_ device recorded (black dashed line, obscured) before charge, (black line) after charging to 0.2 C/cm^2^ and (red line) after 35 minutes discharge through the LED circuit. The initial and discharged spectra are sufficiently overlapping that the black dashed line is not visible.

Figure S10. (A) Variation of transmittance of the FTO/Zn device monitored at 633 nm upon zinc electroplating/electrostripping at 1 mA/cm^2^. The red, blue and black curves correspond to the 1^st^ cycle, recorded in the (red) PEG-free electrolyte for a charge of 0.1 C/cm^2^, (blue) optimized PEG-containing electrolyte for a charge of 0.05 C/cm^2^, and (black) optimized PEG-containing electrolyte for a charge of 0.1 C/cm^2^. For this latter setup, curves corresponding to different cycles are also overlaid (dark to light magenta for cycles 5 to 75). (B) Transparency retention in the bleached state, monitored at 633 nm during the cycling experiments in A (same color code).


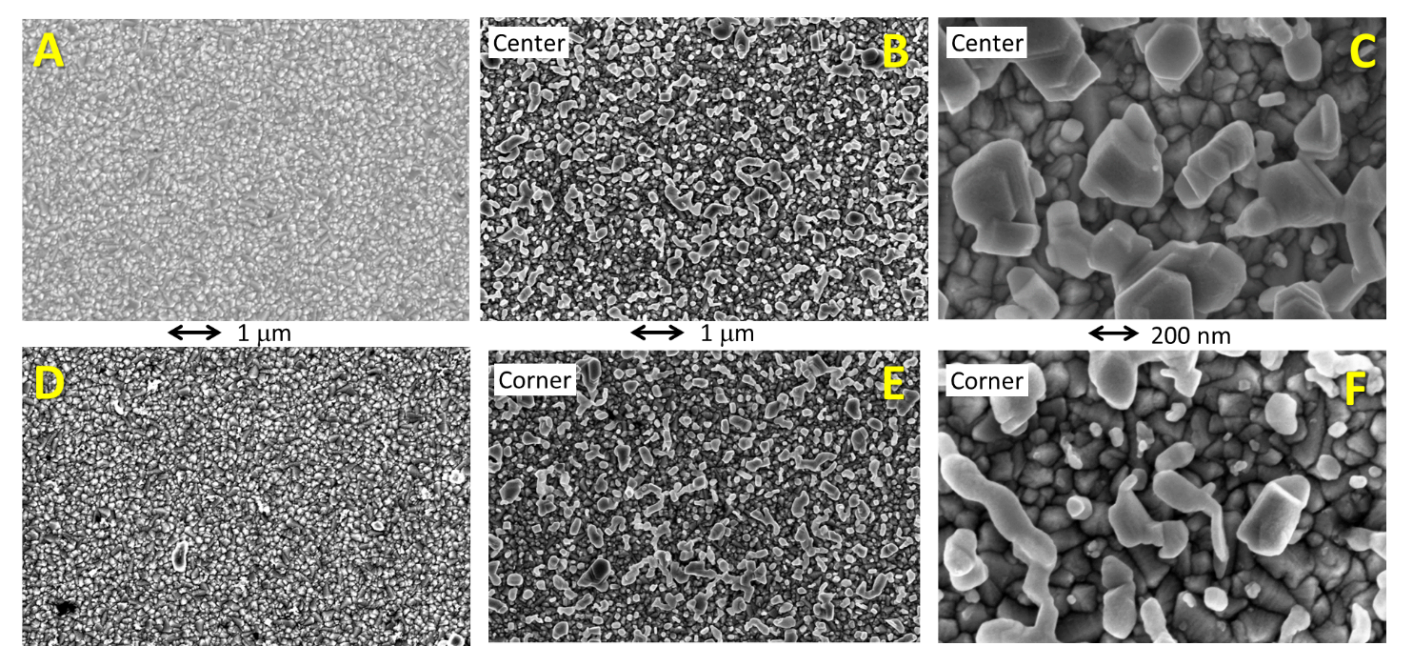


Figure S11. SEM images recorded at FTO electrodes for different states of Zn deposition, on two different zones (center and corner). (A) bare electrode; (B, C, E, F) electrode after Zn electroplating in the optimized PEG-containing electrolyte and (D) electrode after a complete galvanostatic Zn electrodeposition/electrostripping step. Galvanostatic cycling was performed at 1 mA/cm^2^ with a load of 0.1 C/cm^2^.

Figure S12. Galvanostatic cycling of the Zn/ITO_1_ smart window for a charge set at 0.1 C/cm^2^ and a charging rate of 1 mA/cm^2^. The electrolyte was 1 M acetate buffer containing 10% vol. PEG-200 along with 0.4 M Zn(Ac)_2_ and 0.1 M Mn(Ac)_2_ . (A) Galvanostatic charge/discharge potential curves of (black) 1^st^ and (red) 50^th^ cycles. (B) Coulombic efficiency over cycling. (C) Transmittance spectra of the (solid line) discharged and (dashed line) charged device. (D) Transmittance at 633 nm monitored over 30 cycles.


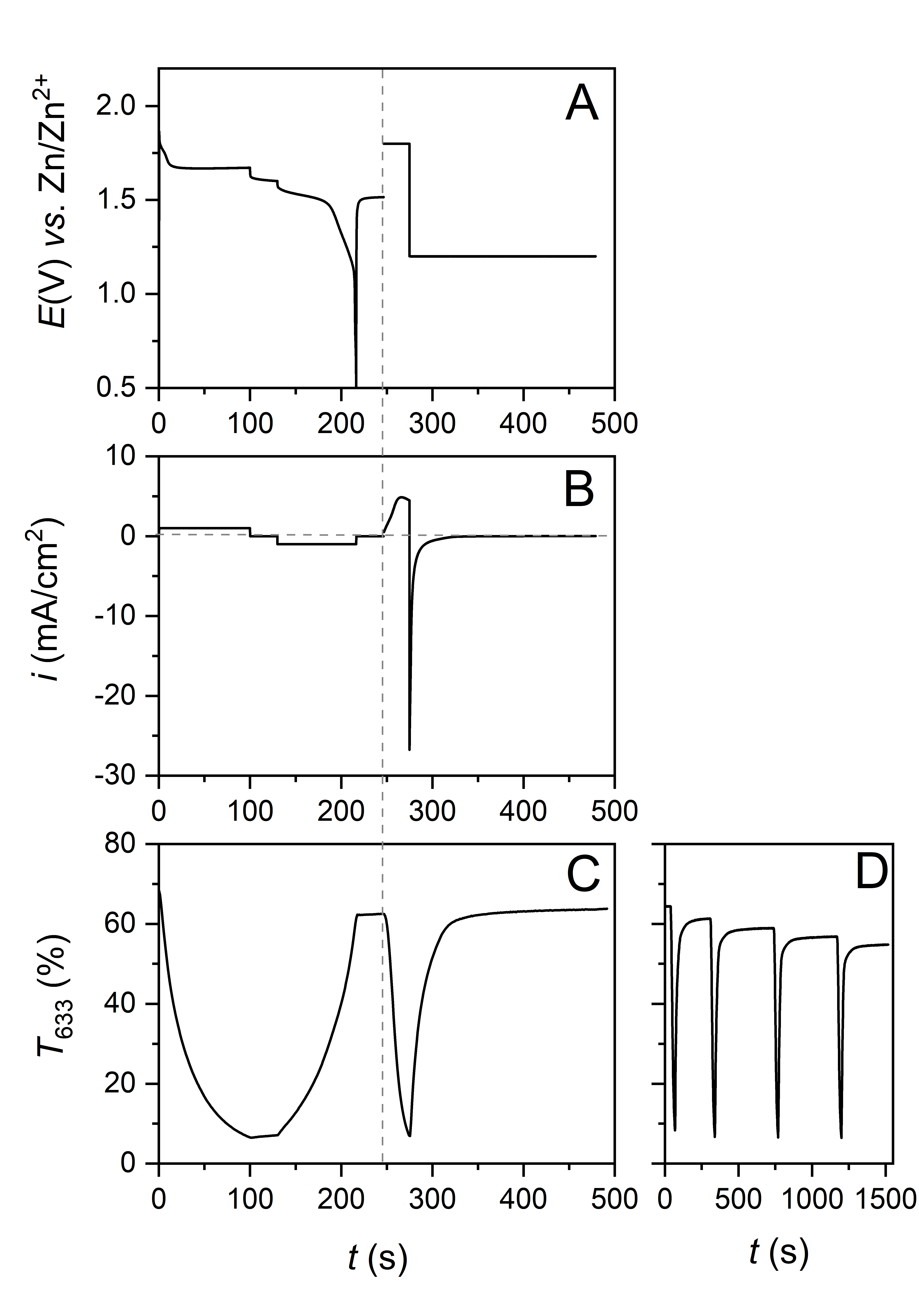


Figure S13. Sequential experiments of a galvanostatic cycle (the cycle is as follow: charge rate of 1 mA/cm^2^ for 100 s + OCP for 30 s + discharge rate of -1 mA/cm^2^ until the cut-off of +0.5 V is reached) followed by a potential-step chronoamperometric cycle (the cycle is as follow: step-potential to 1.8 V until 0.1 C/cm^2^ is passed + step-potential to 1.2 V for 200 s) applied to a Zn/ITO_1_ smart window. The electrolyte was a 1 M acetate buffer containing 10% vol. PEG-200 along with 0.4 M Zn(Ac)_2_ and 0.1 M Mn(Ac)_2_. (A) Potential as a function of time. (B) Current density as a function of time. (C) Transmittance monitored at 633 nm as a function of time. (D) Transmittance monitored at 633 nm as a function of time for the following 4 potential-step chronoamperometric cycles.


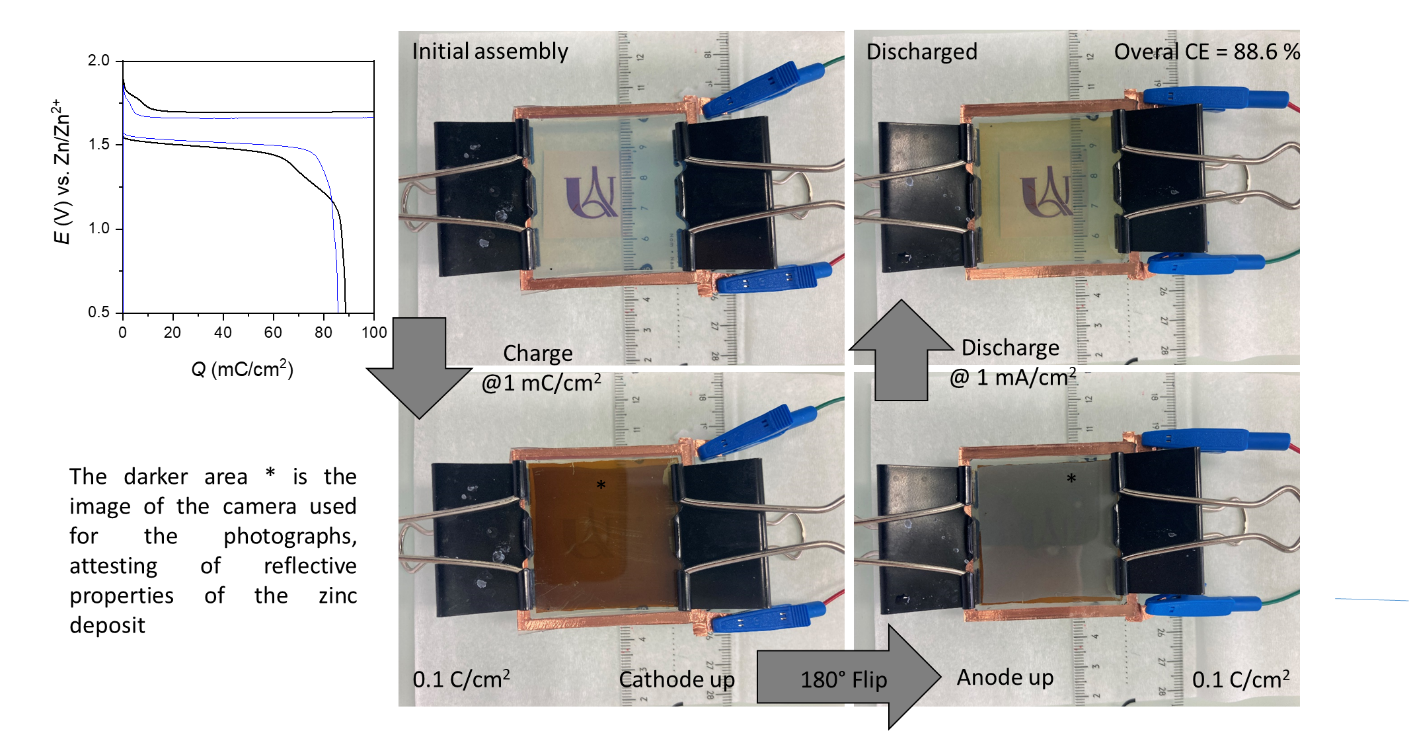


Figure S14. First charge/discharge galvanostatic cycle monitored at a 25 cm^2^ FTO/ITO_1_ smart window, for a charging rate of 1 mA/cm^2^ and a total charge set at 0.1 C/cm^2^. The electrolyte was a 1 M acetate buffer containing 10% vol. PEG-200 along with (black) 0.4 M Zn(Ac)_2_ and 0.1 M Mn(Ac)_2_. Left: first galvanostatic charge/discharge profiles of the (black) 25 cm^2^ and (blue) 6.25 cm^2^ devices. Right: photographs of the 25 cm^2^ device throughout the first cycle, showing successively the device in its initial state, first charged state (for both the cathode and anode sides positioned upwards ), and first discharged state.

Figure S15. Effect of the electrolyte composition on the galvanostatic cycling of FTO/ITO_1_ smart windows for a charge set at 0.1 C/cm^2^ and a charging rate of 1 mA/cm^2^. The electrolyte was 1 M acetate buffer containing 10% vol. PEG-200 along with (black) 0.4 M Zn(Ac)_2_ and 0.1 M Mn(Ac)_2_; (green) 0.5 M Zn(Ac)_2_ and 0.1 M Mn(Ac)_2_ and (red) 0.25 M Zn(Ac)_2_ and 0.25 M Mn(Ac)_2_. (A) Charge/discharge potential curves. (B) Coulombic efficiency over 50 cycles. (C) Transmittance coutinously monitored at 633 nm during cycling.


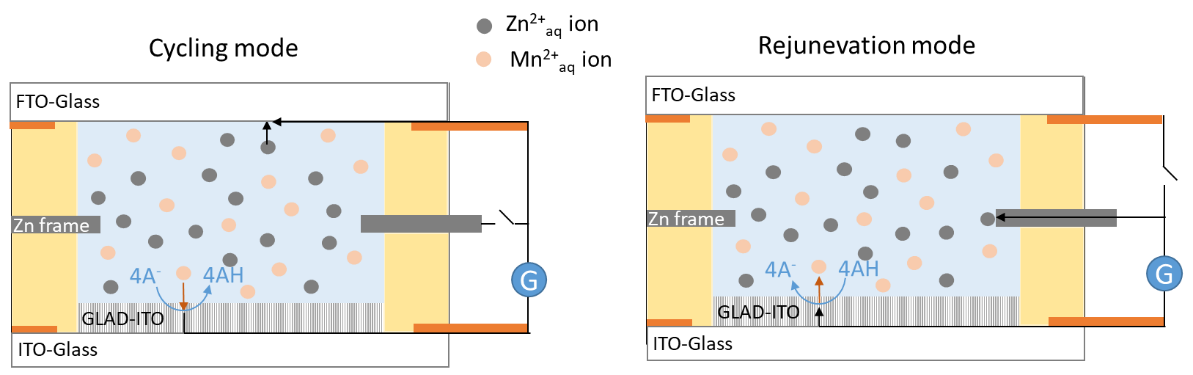


Figure S16. Operating principle of the FTO/Zn/ITO_x_ assembly in the (left) charging and (right) rejuvenation modes.

Figure S17. The two-successive *in-situ* potentiometric curves recorded upon discharging the ITO_1_ cathode *vs*. the sacrificial Zn frame anode in the FTO/Zn/ITO_1_ device after each series of 20 cycles. (Black to orange): first to 5^th^ series, with a discharge intensity of (plain lines) first 1 mA/cm^2^ and (dashed lines) 0.3 mA/cm^2^.
